# Home Health Delivery for Persons With Dementia: Impacts of the New Medicare Payment System

**DOI:** 10.1093/geroni/igaf122.283

**Published:** 2025-12-31

**Authors:** Julia Burgdorf, Tracy Mroz

**Affiliations:** VNS Health, New York, New York, United States; University of Washington School of Medicine, Seattle, Washington, United States

## Abstract

Medicare-funded home health care (HH) is a crucial resource helping Persons with Dementia (PwD) meet their care needs while aging in place. HH includes a mix of skilled nursing, rehabilitation, and personal care services delivered in the patient’s home and one-third of HH patients have dementia. In 2020, Medicare implemented a new HH payment system: the “Patient Driven Groupings Model” (PDGM). PDGM includes several provisions that effectively reduce reimbursement for the types of HH most used by PwD. Using 2019 and 2021 linked Medicare HH claims, patient assessment, geographic, and HH agency data, we modelled the types and intensity of services received as a function of patient dementia status and year (i.e., pre- or post-PDGM). Our sample included older adults (65+) receiving community-initiated HH in 2019 or 2021 (n = 907,553). We modelled outcomes using multivariable logistic and negative binomial regression, adjusting for patient sociodemographic characteristics, functional and clinical status, geographic location, and HH agency characteristics. Compared to pre-PDGM, PwD post-PDGM had lower odds of receiving nursing (Adjusted Odds Ratio (aOR): 0.73; 95% Confidence Interval (CI): 0.72-0.75), occupational therapy (aOR: 0.86; 95% CI: 0.84-0.88), social work (aOR: 0.76; 95% CI: 0.73-0.78), and aide services (aOR: 0.70; 95% CI: 0.68-0.72). Findings persisted following multiple sensitivity analyses, including adjusting for county-level COVID-19 infection rates. This work presents the first analysis of how HH care delivery shifted for PwD following PDGM; findings suggest unintended consequences that may threaten HH quality and the ability to safely age in place for the high-need population of community-living PwD.

